# Identifying cognitive capabilities required for optimal surface extravehicular activity performance

**DOI:** 10.1038/s41526-025-00545-1

**Published:** 2025-12-06

**Authors:** Steven R. Anderson, Mercedes N. Jorge, Suzanne T. Bell

**Affiliations:** 1 KBR, Behavioral Health & Performance Laboratory, Biomedical Research and Environmental Sciences Division, Human Health and Performance Directorate, NASA Johnson Space Center, Houston, TX USA; 2 JES Tech, Behavioral Health & Performance Laboratory, Biomedical Research and Environmental Sciences Division, Human Health and Performance Directorate, NASA Johnson Space Center, Houston, TX USA; 3 National Aeronautics and Space Administration (NASA), Behavioral Health & Performance Laboratory, Biomedical Research and Environmental Sciences Division, Human Health and Performance Directorate, NASA Johnson Space Center, Houston, TX USA

**Keywords:** Human behaviour, Neuroscience

## Abstract

Surface extravehicular activity (EVA) is one of the most cognitively demanding actions that astronauts can execute. Decrements in cognitive performance present an important risk to crew safety, yet there is currently insufficient data to characterize the cognitive capabilities required for optimal surface EVA performance. Here, we conducted a cognitive task analysis with 15 astronauts and subject matter experts to characterize the cognitive demands of surface EVA tasks and to identify the risks associated with decrements in cognitive performance during surface EVA. The information gathered from this study identifies the specific cognitive capabilities that astronauts will need for future surface EVA and provides the foundation for (1) prioritized and targeted cognitive performance measurement, (2) EVA simulation design at varying levels of cognitive workload, and (3) the development of technologies that can inform NASA standards and guidelines and EVA planning for future exploration class missions.

## Introduction

Decrements in cognitive performance present an important risk to crew safety during surface exploration extravehicular activity (EVA). Yet the cognitive capabilities that will be required of crewmembers to maintain crew safety during surface EVA, and the characterization of cognitive workload and cognitive performance around surface EVA is still not well understood. The NASA Human Research Program (HRP) has identified several risks related to injury and compromised performance associated with conducting future surface EVA on the Moon and Mars^1^. Developing a long-term presence on the Moon and exploring Mars will require more frequent and extended EVA compared to EVA conducted during the Apollo era. In addition to important considerations of the physical workload and capacity required to complete surface EVA, there is a need for a commensurate consideration of the cognitive capabilities that will be required to complete the complex science and infrastructure goals of future surface exploration missions.

Research suggests that there are cognitive performance changes during spaceflight; however, these changes are still not well understood^2^. Anecdotal evidence from astronauts reporting “space fog” and other cognitive impacts while in space^3^, as well as evidence from spaceflight and spaceflight analog studies suggests that environmental stressors present during specific mission phases may impact performance in certain cognitive domains. For example, in a recent study of 25 astronauts completing 6-month ISS missions, astronauts demonstrated slower performance in tasks of processing speed, visual working memory, and vigilant or sustained attention in the early flight phase, and a decrease in risk-taking propensity during the late flight and post flight mission phases^4^. Other studies have reported decrements in cognitive domains such as vigilant attention associated with temperature and radiation dose^5^, sleep deprivation^6^, and the post flight mission phase^7^.

In contrast, to date fewer studies have examined cognitive workload and cognitive performance in the context of EVA. Although it is expected that many of the same cognitive capabilities required of astronauts during flight would also be important during surface EVA, there are unique risks to astronaut behavioral health and performance during the high-frequency surface EVA operations planned for future Artemis missions. The physical and cognitive demands of Artemis mission surface exploration EVA are likely to be greater than lunar EVA conducted during the Apollo era^8^ and current microgravity EVA conducted from the ISS.

Evidence attesting to the demands of EVA includes data from the Mars Desert Research Station, in which surface EVA resulted in increased cognitive and physical workload^9^. Novel gait patterns associated with partial gravity, such as during surface exploration EVA on the Moon or Mars, was found in an EVA simulation study to increase cognitive workload and result in increased demands on prefrontal cortex activity and reduced memory recall^10^. In simulated EVA conducted in water submersion, response accuracy during inhibition and switching cognitive tasks was lower during EVA compared with a control condition, while reaction times for the inhibition task were faster during EVA^11^. In a small-sample simulated EVA study conducted in NASA’s Active Response Gravity Offload System (ARGOS), researchers reported a trend suggesting decrements in vigilant attention and processing speed following two simulated EVAs conducted over a three-day period^12^.

Despite these preliminary findings, there remains a need to update the current level of knowledge primarily based on conducting EVA in microgravity from the ISS toward not only a combination of a surface EVA environment but also a significant increase in EVA frequency, which may infer increased risk as well as opportunities for adaptation. Furthermore, it is still unclear which cognitive domains are most important for conducting mission critical decisions with crew safety implications during surface EVA. To address this gap in our understanding, in the present study we conducted an applied cognitive task analysis (CTA) to characterize the procedures, cognitive demands required, and critical safety incidents related to decrements in cognitive performance during surface exploration EVA. Identifying the specific cognitive demands that astronauts are likely to encounter during surface exploration EVA will importantly provide the foundation for: (1) prioritized and targeted cognitive performance measurement and functional performance tests, (2) EVA simulation design at varying levels of cognitive workload, and (3) the development of training and other technologies that can inform NASA standards and guidelines for medical operations and EVA planning in future Artemis missions to the Moon and Mars.

## Methods

A mixed-methods applied CTA^13^ was conducted from July to September 2024. There were two interview protocols, with different sets of subject matter experts (SMEs) interviewed for each protocol to maximize the amount of content that could be covered given our goal of limiting interviews to 1-hour in duration. Each interview was led by a PhD-level scientist in the NASA Behavioral Health & Performance Laboratory with expertise in health psychology and cognitive neuroscience (first author). Notes were taken by a Master’s-level research coordinator in the BHP Laboratory with expertise in industrial-organizational psychology (second author). Interviews were recorded on Microsoft Teams to assist with notetaking.

### Subject matter experts

A total of 15 SMEs at NASA Johnson Space Center (JSC) and Ames Research Center (ARC) participated in the applied CTA interviews. Inputs were sought from experts of different EVA experience levels and occupations. Interviewees comprised 6 experts in EVA research (4 Human Performance Engineers, 1 Surface Mobility Test Team Lead, 1 Crew Health and Performance Officer), 5 experts in EVA operations (2 EVA Flight Controllers, 2 EVA Instructors, 1 Scientist), and 4 astronauts (3 NASA Astronauts, 1 ESA Astronaut).

### Defining EVA parent tasks and subtasks

Interviews were structured around surface exploration EVA parent tasks and subtasks defined through a previous EVA task analysis conducted in the Human Health & Performance (HHP) Directorate at JSC. The EVA task analysis was based on internal Artemis concept of operations (ConOps) documents and a NASA technical manual abridgement^14^ of the Generalizable Skills and Knowledge for Exploration Missions (NASA/CR-2018-22045) report^15^. Conducted under Cooperative Agreement 80NSSC18K0042 for the NASA Human Factors and Behavioral Performance Element in the Human Research Program (HRP), the goal of the technical manual was to identify the tasks that future crew will be conducting on missions to Mars, as well as the knowledge, skills, and abilities that will be required to complete these exploration mission tasks. All EVA parent tasks and subtasks defined in the EVA task analysis were used for the applied CTA. Parent tasks defined in the task analysis as part of contingency EVA were discussed specifically in the EVA Simulation Scenario in Interview 2.

### Interview 1 protocol

The first interview protocol was completed in separate 1:1, 1-hour sessions by 9 SMEs (3 experts in EVA research, 2 experts in EVA operations, 4 astronauts). All astronauts were chosen to complete Interview 1 due to the usefulness of firsthand operational EVA experience in answering this interview’s questions.

Experts were first asked about the specific tasks and cognitive demands associated with surface exploration EVA. This informed a task diagram, which provided a high-level overview of the steps involved in the major tasks conducted during surface EVA, as well as which of the steps require the most cognitive skill. Experts ranked the 11 EVA parent tasks in order of their level of expertise or familiarity with the task (1 = Most Familiarity/Expertise) and in order of relative cognitive demand (1 = Most Cognitively Demanding).

Experts were then asked to provide a rating of cognitive demand for 32 EVA subtasks. Ratings were adapted from the NASA-TLX Mental Demand dimension^16^. How much cognitive and perceptual activity do you think would be required for effective performance of this subtask? (0 = Low, 100 = High). Experts could also choose to define their own subtasks based on their expertise. There were several goals in obtaining quantitative cognitive demand ratings for surface EVA parent tasks and subtasks: (1) to characterize EVA tasks at both the parent and subtask level, (2) to facilitate comparison between different surface EVA parent tasks and subtasks, and (3) to provide a quantitative basis by which cognitive demand ratings elicited in future EVA studies could be compared given the lack of available quantitative data on the cognitive demands of surface EVA tasks (particularly those that are not typically included in EVA simulations).

Next, experts completed a knowledge audit, which employed a set of probes designed to describe types of domain knowledge of skills and to elicit appropriate examples. The content of the knowledge audit was adapted from the applied CTA methodology^13^. and was designed to elicit concrete examples in the context of surface EVA, cues and strategies used, and why specific EVA tasks are cognitively demanding. The knowledge audit focused on the EVA parent tasks with which each expert indicated they had the highest level of expertise. Experts who had not completed a real or simulated EVA firsthand were asked to respond to knowledge audit probes based on their experience observing EVA completed by others. There were eight probes per parent task: Past and Future, Big Picture, Noticing, Job Smarts, Opportunities/Improvising, Self-Monitoring, Anomalies/Off-Nominal Situations, and Equipment/Spacesuit Difficulties. Expert responses to the series of knowledge audit probes (e.g., Can you give me an example of what is important about the Big Picture for EVA Prep/Post Ops?) were categorized into “Aspects of Expertise,” “Cues and Strategies,” and “Why Difficult?” per the categories suggested by Militello and Hutton (1998).

### Interview 2 protocol

The second interview protocol was completed in separate 1:1, 1-hour sessions by 6 SMEs (3 experts in EVA research and 3 experts in EVA operations).

Experts were asked to first describe the events that would be completed as part of an Incapacitated Crew Rescue (ICR) scenario^17^. In the scenario communicated to experts, developed for this interview, an extravehicular crewmember (EV1) has strained their back while conducting field geology 2 km away from a landing vehicle and has become incapacitated on the surface of the Moon, requiring rescuing by the other crewmember (EV2). Experts were asked to imagine that they were the rescuing crewmember on the Moon in the incident (EV2) and to list the major events that would characterize their response in this situation (e.g., initial response, contingency walk back to landing vehicle, communication with Mission Control Center [MCC]). Expert response to the simulation scenario were categorized as “Events,” “Actions,” “Assessment,” “Critical Cues,” and “Potential Errors.”

Next, experts were asked to generate knowledge, skills, and abilities (KSAs) underlying each EVA parent task. The linkage analysis focused on the parent tasks with which each expert indicated they had the highest level of expertise. To aid experts in brainstorming KSAs, we defined KSAs as Knowledge: Body of information, can be fact-based or from practical experience; Skills: Developed capacities, either deliberately learned or as a byproduct of task performance; Abilities: Enduring capacities, bound by innate capability or capacity (e.g., balance, endurance, coordination). Experts were asked to define as many KSAs as needed for each parent task.

**Fig. 1:**
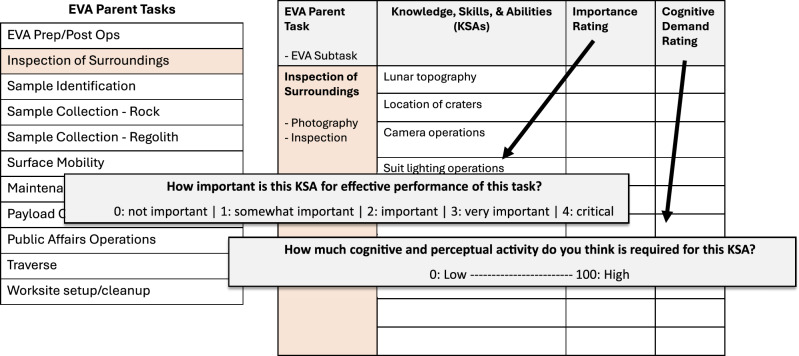
Linkage analysis.

Experts then made ratings of importance and cognitive demand for each KSA identified (Fig. 1). The Importance Rating was adapted from a prior job analysis^18^ and asked: How important is this KSA for effective performance of this task? (0 = Not Important, 1 = Somewhat Important, 2 = Important, 3 = Very Important, 4 = Critical). The Cognitive Demand Rating was adapted from the NASA-TLX Mental Demand dimension^16^ and asked: How much cognitive and perceptual activity do you think would be required for effective performance of this KSA? (0 = Low, 100 = High).

Ratings of importance and cognitive demand for KSAs were used to guide the subsequent cognitive domain mapping step, in which cognitive domains and their putative neural correlates were linked to KSAs to gain insight into the deeper cognitive capabilities required for surface EVA tasks.

### Closing questions

At the end of both Interview 1 and Interview 2, all experts were asked two additional questions to understand factors that could contribute to cognitive workload during surface EVA. First, they were asked to list what they thought were the most likely and consequential critical safety incidents related to decrements in cognitive performance that could occur during future exploration EVA on the Moon and Mars. Next, experts were asked to provide feedback on how a lunar communication delay might impact an astronaut’s cognitive performance on future Artemis missions. Both closing questions were intended to fill in gaps and address relevant topics to surface EVA that may not have been addressed earlier in the interviews.

Steps taken to address the potential for response bias among experts included the inclusion of subject matter experts of different EVA experience levels and occupations, the use of standardized rating scales, the use of consistent rating scale anchors, and training experts how to use the rating scales during each 1-hour interview. All cognitive demand ratings and rankings are presented by SME occupation (astronaut, EVA operations, EVA research) to account for differences in background that might influence ratings. Notes from each interview were verified against recordings where needed, then synthesized based on the specific goals of each interview section. Themes were extracted from the notes from each interview and compiled into a knowledge audit, simulation scenario, and cognitive demands table to summarize results across all interviews and extract insights. Ratings were analyzed using R Studio Version 4.4.1.

## Results

### EVA experience and expertise

**Fig. 2:**
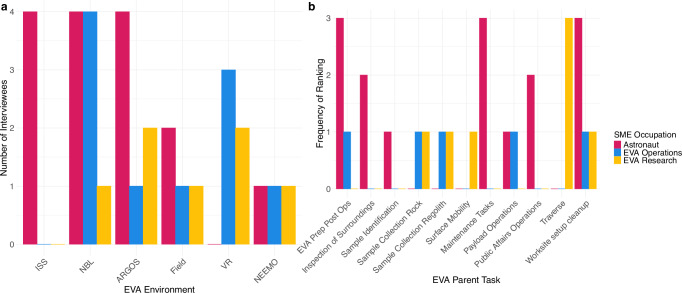
EVA experience and expertise. **a** Subject matter expert (SME; *N* = 15) level of experience by EVA environment and SME occupation. **b** Frequency of familiarity/expertise rankings of surface exploration EVA parent tasks by SME occupation. Astronaut (*n* = 4), EVA Operations (*n* = 5), and EVA Research (*n* = 6) experts were asked to rank EVA parent tasks in terms of their personal level of familiarity or expertise (1 = Most Familiarity/Expertise). Rankings below 1 were excluded for visual readability. ISS International Space Station, NBL Neutral Buoyancy Laboratory, ARGOS Active Response Gravity Offload System, Field Field Test, VR Virtual Reality, NEEMO NASA Extreme Environment Mission Operations.

Experts had an average of 7.5 years (range = 2–19, SD = 5.3) of EVA experience across multiple spaceflight and spaceflight analog settings (Fig. 2a). Experts had the most experience with EVA in Neutral Buoyancy Laboratory (NBL), Active Response Gravity Offload System (ARGOS), Virtual Reality (VR), and International Space Station (ISS) environments. Astronauts had a total of 71 h 33 min of EVA time on ISS. In rankings of familiarity/expertise (Fig. 2b), experts reported a spread of expertise across the EVA parent tasks, and most often ranked the following parent tasks as highest (#1) in expertise: Worksite Setup/Cleanup (5 experts), EVA Prep/Post Ops (4 experts), Maintenance Tasks (3 experts), and Traverse (3 experts).

### Cognitive demand rankings

**Fig. 3:**
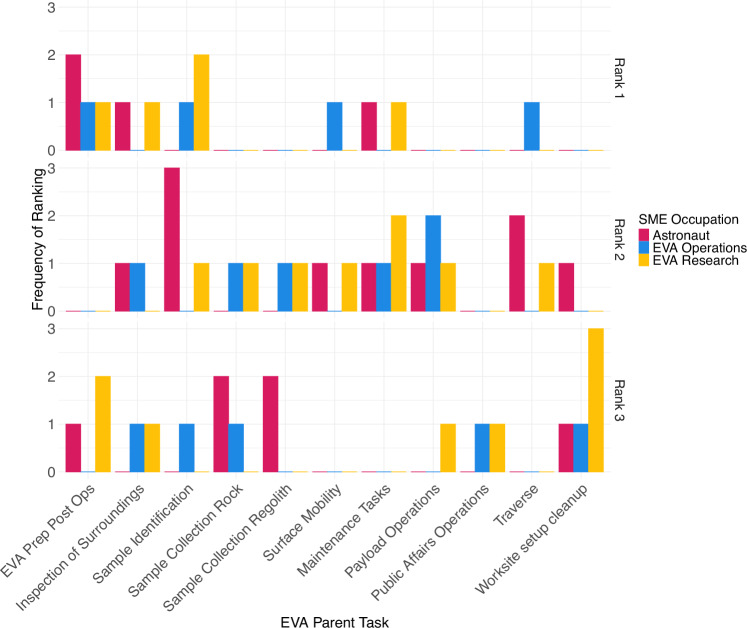
Cognitive demand rankings of surface EVA parent tasks. Subject matter expert (SME; *N* = 15) rankings of cognitive demand of surface EVA parent tasks by SME occupation. Astronaut (*n* = 4), EVA Operations (*n* = 5), and EVA Research (*n* = 6) experts were asked to rank surface EVA parent tasks in terms of cognitive demand (1 = Most Cognitive Demand). Rankings below 3 were excluded for visual readability.

Experts were asked to rank the EVA parent tasks in terms of how much cognitive and perceptual activity is required for effective performance of that task. Experts indicated that perceived cognitive demand was distributed across multiple parent tasks (Fig. 3) and ranked the following parent tasks as highest (#1) in cognitive demand: EVA Prep/Post Ops (4 experts), Sample Identification (3 experts), Inspection of Surroundings (2 experts), and Maintenance Tasks (2 experts).

### Cognitive demand ratings

Experts in Interview 1 and 2 completed cognitive demand ratings at different levels of analysis of EVA tasks (i.e., subtask-level and KSA-level) to provide a more granular characterization of the cognitive demands of EVA task components. To examine cognitive demand for EVA parent tasks across the full sample of experts interviewed (*N* = 15), cognitive demand ratings for each EVA subtask (nested within parent task) were combined from experts in Interview 1 (*n* = 9) with the cognitive demand ratings for each KSA (nested within parent task) provided by experts in Interview 2 (*n* = 6).

**Fig. 4:**
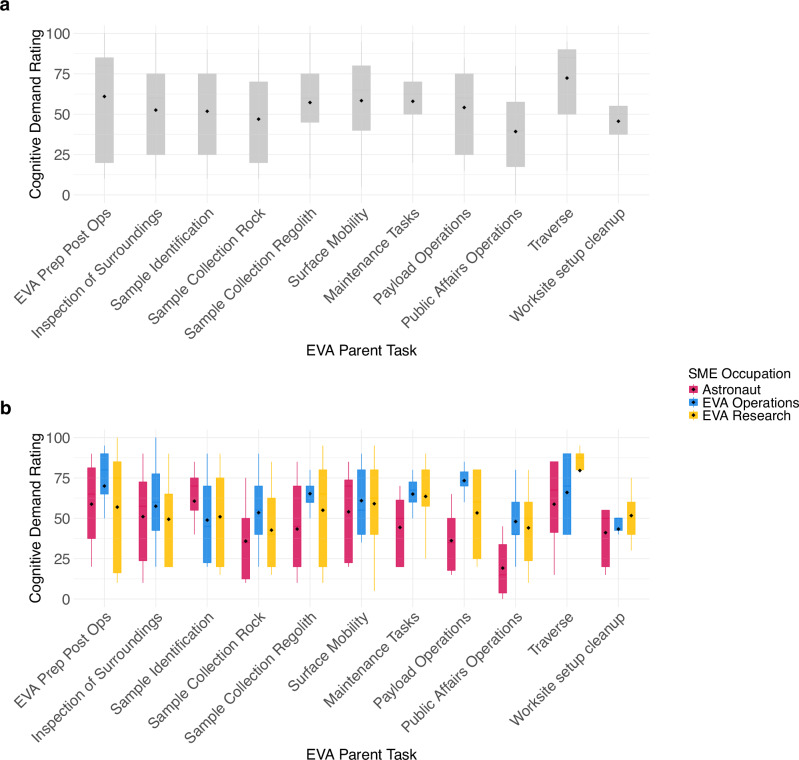
Cognitive demand ratings of surface EVA parent tasks. **a** Subject matter expert (SME; *N* = 15) ratings of cognitive demand by EVA parent task; **b** SME (*N* = 15) ratings of cognitive demand by EVA parent task and SME occupation. Cognitive demand ratings were combined across all SMEs in both interviews for each parent task. Parent task ratings were combined from the subtask ratings in Interview 1 (*n* = 9) and the knowledge, skills, and abilities (KSA) ratings in Interview 2 (*n* = 6) that were made within each parent task. All ratings are presented as boxplots with diamonds representing means, lines representing medians, and whiskers representing upper and lower quartiles.

Across the full sample of experts interviewed (*N* = 15), the following EVA parent tasks were rated as having the highest cognitive demand. Traverse (M = 72.4, SD = 24.2), EVA Prep/Post Ops (M = 61.0, SD = 30.9), Surface Mobility (M = 58.4, SD = 26.3), Maintenance Tasks (M = 58.0, SD = 20.9), and Sample Collection: Regolith (M = 57.2, SD = 25.5) (Fig. 4a). Examining cognitive demand ratings by SME occupation (Fig. 4b), astronauts rated the surface EVA parent tasks as overall less cognitively demanding (M = 46.8, SD = 25.9) than EVA operations (M = 59.0, SD = 22.0) and EVA research (M = 55.3, SD = 27.0) personnel. The parent task rated as most cognitively demanding by astronauts was Sample Identification (M = 60.6, SD = 24.6). In contrast, EVA operations personnel rated Payload Operations as the most cognitively demanding task (M = 73.3, SD = 8.76). EVA research personnel indicated that Traverse was the most cognitively demanding task (M = 79.6, SD = 19.7). Cognitive demand ratings for all EVA parent tasks are available in Supplementary Table 1.

Among experts (*n* = 9) who provided cognitive demand ratings of EVA subtasks in Interview 1, the following subtasks were rated as having the highest cognitive demand: Suit checks and procedures (solo) (M = 92.5, SD = 3.54), Prebreathe (M = 90, SD = 0), Depressurization/repressurization (Rating = 90), Traverse (in darkness) (M = 90, SD = 0), and Geological Description (M = 88.3, SD = 2.89). Subtask ratings without descriptive statistics were provided by a single expert. Cognitive demand ratings for all EVA subtasks are available in Supplementary Table 2.

Among experts (*n* = 6) who generated and provided cognitive demand ratings of KSAs in Interview 2, the following KSAs were rated as having the highest cognitive demand: Knowledge of consumables rates (Rating = 95), Knowledge of mission operations, briefing, lunar topography, and hazard analysis (Rating = 95), Navigation skills (Rating = 95), Describing geology (Rating = 90), and Ability to manage secondary tasks (navigation, monitoring consumables) while prioritize primary tasks (Rating = 90). KSAs without descriptive statistics were provided by a single expert. Cognitive demand ratings for all KSAs are available in Supplementary Table 3.

### Knowledge audit

Several key points and themes emerged from the knowledge audit across EVA parent tasks. Specific tasks that were highlighted as cognitively demanding included EVA Prep Ops, which experts reported as one of the most safety critical tasks an astronaut can execute, with the cognitive demand higher for the support intravehicular (IV) compared to extravehicular (EV) crewmember. Experts also highlighted navigation during traverse on the lunar surface as challenging and safety critical. Challenges during navigation include the visually similar landscape, lack of landmarks, low illumination, and permanently shadowed regions (PSRs). Geological sample identification was also noted as a highly cognitively demanding task. Challenges mentioned by experts during this task included remembering the scientifically precise geological terminology, being able to visually identify subtle differences in samples in low lighting conditions and identifying samples accurately to the ground science team while also managing consumables, communication, navigation, and suit mobility.

Themes that emerged as relevant for cognitive performance across EVA tasks included (1) decision-making during EVA that simultaneously balances safety, time constraints, and mission objectives, and (2) situational awareness (SA) as a key target of cognitive workload. Factors that experts indicated increase SA include sufficient ground/IV support, sufficient sleep, clear visuals on the environment, and manageable physical workload. In contrast, factors that decrease SA included insufficient ground/IV support, fatigue due to insufficient sleep, poor visuals on the environment (e.g., due to shadows, bright sunlight, or other obstruction), overly fixating on an off-nominal signal, and high physical workload (e.g., pushing a heavy tool cart up a steep incline). High physical workload was highlighted as a major factor that can increase cognitive workload. EVA is physically demanding, and the physical demands of the overall EVA may increase cognitive workload independent of cognitive task demands of a specific task. For example, cognitive resources could be depleted as the crewmember focuses their effort on managing the presence of any pain or hotspots.

Experts mentioned several factors that could help reduce cognitive workload during surface EVA. One included effective teamwork: a good teammate can offload cognitive workload, while a suboptimal teammate (e.g., due to incompatible working or learning styles) can increase cognitive workload. In addition, experts mentioned that it is critical to have extensive pre-mission training and well-written procedures that crewmembers can effectively reference during the mission. Human factors considerations can help design procedural checklists that are easy to navigate (e.g., by having a touchscreen checklist with a color indicating current step).

### Simulation scenario

The results of the ICR simulation scenario were summarized into the following sequence of high-level events identified by experts: (1) Determine injured crewmember status, (2) Check ICR procedures, (3) Communicate with MCC and IV crewmember, (4) Secure worksite, (5) Configure ICR transport device, (6) Secure injured crewmember to ICR transport device, (7) Traverse back to lander with injured crewmember. A description of the summarized actions, assessments, critical cues, and potential errors for each event follows.

To determine injured crewmember status, the major actions experts identified were for the EV to traverse to the injured crewmember if separated, ask what happened, if they can walk, and how much pain they are experiencing. Assessments focused on whether the injured crewmember reports that they cannot walk and if they report a high level of pain. Critical cues included the injured crewmember’s body position, degree of mobility, pain facial expression, and verbal report of pain. Potential errors included underestimating the injured crewmember’s verbal or nonverbal pain indication or failing to address a more urgent issue if present (e.g., suit puncture).

Checking ICR procedures included the action of the EV referring to their cuff checklist for correct ICR procedures. Assessments included the level of specificity determined in the cuff checklist, limitations of what procedures can be accomplished in this scenario, and the level of crew autonomy to make decisions independent of MCC. Critical cues were making sure all individuals (EV, IV, MCC) agreed on the ICR procedures required for each specific situation and ensuring everyone was referring to the same procedures. Potential errors were the EV choosing the wrong cue card or wrong procedures for the specific ICR scenario, a disconnect between which procedures EV and MCC are referencing, and overreliance on cue cards to the detriment of critical environmental cues.

Communicating with MCC and the IV crewmember had actions for the EV to report conditions, actions, and needs to MCC, listen for further instructions from MCC, and update the IV on the situation so that the IV can go ahead and configure the airlock for return. The flight surgeon then develops a plan, making a recommendation while the rest of the team assesses the impact of that recommendation on the timeline and science objectives. Critical cues included instructions from MCC/flight surgeon and the clarity of communication given lunar communication delay, while potential errors included miscommunication, providing too much or too little detail, not providing enough SA to MCC, and assuming MCC had knowledge of critical information even when they do not (e.g., due to potential shift changes over the course of an EVA).

Securing the worksite involved the EV salvaging as much science objectives of the EVA as possible while prioritizing the safety of the injured crewmember. Assessments were to confirm all tools and samples are present and prioritize retrieving samples over tools if it is the last EVA of the mission. Critical cues included the location of tools and samples in the worksite at the time of injury and MCC guidance on the status of science objectives and remaining timeline. A potential error was if the injury event occurred early in the mission, tools left behind at the worksite could preclude accomplishing further science objectives.

Configuring the ICR transport device involved de-configuring the transport device if the current configuration is not ICR compatible and moving the device to a flat surface if on an incline. Assessments indicated that if the device is on an incline, it may not be safe for the injured crewmember. Thus, it is important to determine whether the transport device is undamaged and has all equipment (e.g., straps) needed for securing the injured crewmember. Critical cues were the flatness of the lunar landscape where injury has occurred and the presence of rocks, craters, or other obstacles in the intended return traverse path. Potential errors were improperly de-configuring the ICR transport device and failure to stabilize the transport device before loading injured crewmember.

Securing the injured crewmember to the ICR transport device involved the action of assisting the injured crewmember onto the ICR transport device; if not capable, strap the crewmember and haul onto the device. Assessments were whether the injured crewmember is securely fastened to the ICR transport device and which direction the injured crewmember is facing (as this impacts the EV’s visual on the injured crewmember’s facial expressions). Critical cues included the injured crewmember’s level of mobility and location of straps and other ICR-assistive equipment. Potential errors were failing to properly secure the crewmember to transport device (risking further injury) and missing a step in the ICR procedures. Experts noted that an off-nominal scenario means there is likely less training and “muscle memory” to rely on.

Traversing back to the lander with the injured crewmember involved asking MCC if there are any hazards on the route back to the lander, looking at the map in conjunction with MCC instruction, walking forward with the injured crewmember either being pushed or pulled, and building in waypoints for checking on navigation, consumables, and the injured crewmember’s status during the return route. Assessments were to identify the flattest and least hazardous return traverse path possible (i.e., free of craters, PSRs, boulders) and to determine a return path that both the EV and MCC agree upon. Critical cues were the level of consumables remaining for both crewmembers, unexpected hazards on the traverse route, the slope of the traverse route, and the lighting of the traverse route (avoiding shadows, as they may conceal hazards). There were multiple potential errors identified for this event. They included not factoring in the rate of change of consumable due to the emergency scenario, traversing on a slope without realizing it, choosing a traverse path that initially seems like a shortcut but that includes a steep slope on the other side of an incline, and losing track of current location and getting lost.

### Linkage analysis

As part of the linkage analysis, experts provided a rating of how important each KSA generated was for effective performance of each EVA parent task (0 = Not Important, 1 = Somewhat Important, 2 = Important, 3 = Very Important, 4 = Critical). Although KSAs were overall rated as highly important given that they were generated by the experts rating them, there was nevertheless variability among ratings. KSAs provided for Traverse (M = 3.33, SD = 0.71), Sample Collection: Regolith (M = 3.27, SD = 0.47), and EVA Prep/Post Ops (M = 3.20, SD = 0.86) were rated as highest in importance by experts.

**Fig. 5:**
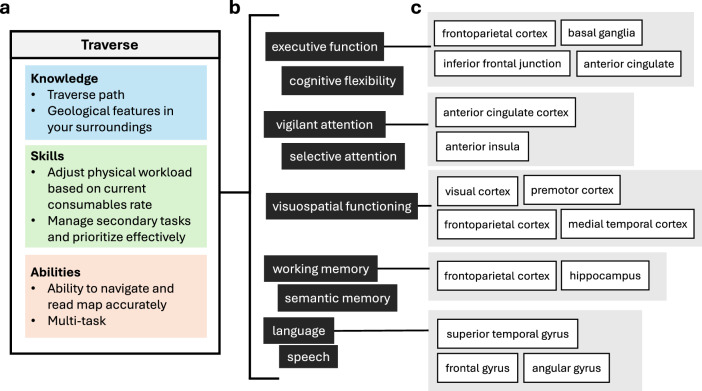
Cognitive domain mapping for surface EVA traverse. **a** Summary of knowledge, skills, and abilities (KSAs) identified by subject matter experts (*n* = 6) for surface EVA traverse; **b** Cognitive domain mapping of KSAs; **c** Summary of neural correlates of identified cognitive domains of KSAs.

For the cognitive domain mapping step of the linkage analysis, we focused on the three EVA parent tasks whose KSAs were rated as highest in cognitive demand: Traverse (M = 88.3 SD = 5.59), Maintenance Tasks (M = 83.3, SD = 5.16), and Sample Collection: Regolith (M = 61.4, SD 13.4). We then linked them to their underlying cognitive domains and putative neural correlates suggested by published literature (Traverse depicted in Fig. 5).

Cognitive domains identified for Traverse KSAs (e.g., knowledge of geological features in path, skill to manage consumables, ability to read map) included executive function (involved in problem solving and conceptual understanding), cognitive flexibility (an aspect of executive function concerning the adaptability of cognitive processing strategies to face new and unexpected conditions in the environment^19^), vigilant attention, visuospatial functioning, working memory, language, and speech. Neural correlates of these cognitive domains include frontoparietal and cerebellar regions involved in executive function^20^; lateral and orbital frontoparietal, midcingulo-insular and frontostriatal regions involved in cognitive flexibility^21^; frontoparietal, anterior insula, thalamic and cerebellar regions involved in vigilant attention^22^; visual cortex, prefrontal, premotor, and medial temporal regions identified in visuospatial processing^23^; frontoparietal, cerebellar, and subcortical regions implicated in working memory^24^; and inferior frontal gyrus and temporal cortex regions involved in speech production and language comprehension^25^.

For Maintenance Task KSAs (e.g., knowledge of how tools and equipment work, skill to use tools to complete repairs on hardware, ability to plan ahead and manage time), cognitive domains identified included executive function, cognitive flexibility, attention, language, speech, working memory, gross motor skills, and fine motor skills. Neural correlates of these cognitive domains include the brain regions underlying executive function, cognitive flexibility, attention, working memory, and speech and language identified above, in addition to motor cortex and cerebellar regions implicated in the gross and fine motor control^26^ required to complete complex maintenance tasks.

For Sample Collection: Regolith KSAs (e.g., knowledge of geology terminology for geological callouts, the skill to extract a drive tube sample, and the ability to physically drive tube into lunar soil), cognitive domains identified included similar executive function, cognitive flexibility, attention, language, speech, working memory, and motor skills domains as Maintenance Tasks. Accordingly, the neural correlates for Sample Collection: Regolith would be largely overlapping with Maintenance Tasks.

### Closing questions

Experts were asked what they thought were the most likely and consequential critical safety incidents related to decrements in cognitive performance that could occur on future surface EVAs on the Moon and Mars. Experts identified several different scenarios of concern. One of the most recurring comments concerned off-nominal and emergency scenarios. Experts noted that these scenarios will be highly cognitively demanding for crew and that one’s brain can seem to “shut down” during high-pressure situations, which can make it difficult to process new or less familiar information. This is especially true if crew are already fatigued due to high physical workload or sleep decrements.

Errors in navigation during traverse were also highlighted, with experts noting that getting lost on the Moon is a life-threatening scenario. Navigation during traverse will be challenging due to high-contrast shadows and the visually similar lunar landscape, so any fall could lead to injury, necessitating ICR. The need to muti-task and track consumables while also driving a rover will add further cognitively challenging elements to navigation. Experts noted that errors following procedures, such as missing procedural steps, will increase as the crew becomes more physically and cognitively fatigued, especially if traversing without a rover and engaging in high-tempo EVA cadence.

Sleep was another key theme mentioned in this section. Prior research has found that inadequate sleep due to new vehicles and the complexity of mission phases (e.g., orbit, landing) leading up to surface EVA could have multiple downstream negative effects on cognitive and physical performance^27^. Errors in donning and doffing the suit due to lack of sleep and increased fatigue would be detrimental to crew safety and science objectives. Decrements in SA due to fatigue could lead to neglect of consumables monitoring, while decrements in attention to detail due to fatigue could lead to losing track of details with crew safety implications. Finally, incomplete dust mitigation and inspection due to post-EVA fatigue could result in cumulative hazardous lunar dust exposure over time.

Experts were also asked how they thought a 5–12 s lunar (voice) communication delay might impact an astronaut’s cognitive performance on future Artemis missions. The majority of experts agreed that lunar communication delay is an area of concern that needs further characterization and research. Experts noted that lunar communication delay would likely lead to repetition and possible talking over each other, potentially causing frustration and annoyance among crewmembers and ground support. Mood states such as frustration and annoyance are known to impact cognitive performance due to the shared neural substrates of emotion and cognition^28^. Consistent with responses to our previous question, experts noted that off-nominal and emergency scenarios will make any communication delay especially impactful as crewmembers will need to communicate with MCC in a time-pressured and stressful situation. Finally, experts noted that there is a pressing need to develop guidelines for when and how to stop speaking to clarify communications. Procedure words used in military and aviation settings (e.g., “over”, “copy”) should be worked into EVA ground simulations and training and used consistently to prepare crew for future communication delay scenarios. Communication between crew and MCC in future missions may be different than it currently is on ISS. The expectation that crew will be in near-constant communication with MCC may not be realistic during Artemis missions and could contribute to increased cognitive workload for crewmembers.

### Cognitive demands table

The final output of the CTA was a cognitive demands table (Table 1) which acts as a synthesis of the information gathered in all interviews conducted. The table is organized into difficult cognitive elements and lists the challenges, cues and strategies, and cognitive domains associated with each element.

**Table 1:** Cognitive demands table

Difficult Cognitive Element	Why Difficult	Cues and Strategies	Cognitive Domains
Maintaining situational awareness (SA)	- Fatigue - Large amounts of information in environment	- Support from MCC/IV - Knowing when you can conserve cognitive resources	- Executive function - Vigilant attention - Selective attention
Navigation during traverse	- Lack of visually distinct landmarks on Moon, low illumination, long shadows, few navigational aids - No perfect ground analog for partial gravity EVA to practice	- Good maps and pre-brief materials - Frequent communication with MCC/IV to confirm location - Handrails, backstops, waypoints, and landmarks to orient	- Executive function - Cognitive flexibility - Working memory - Language - Speech - Visuospatial functioning - Reading comprehension
EVA Prep Ops	- Critical procedure; error could endanger lives of crewmembers - Large number of procedural steps - Requires effective teamwork	- Aim to be ahead of schedule; easier to slow tempo than speed up - Formulate and discuss plan the night before - Call out procedure steps while completing them	- Executive function - Working memory - Vigilant attention - Language - Speech - Reading comprehension
Managing safety, time constraints, and mission objectives simultaneously over entire course of EVA	- Requires decision-making and cognitive flexibility on relative importance of time management, safety, and mission objectives	- Safety is a tool for successful task completion - MCC can offload some of the cognitive load of managing time, safety, and mission objectives	- Executive function - Cognitive flexibility - Vigilant attention - Selective attention - Inhibitory control
Remembering geological terminology and accurately applying it to samples	- Terminology can be complex and specific to this scientific discipline - Astronauts have diverse backgrounds and may not be geologists or scientists by training - Lunar rocks are visually similar and geologically significant differences may be subtle - Geological callouts must occur while multi-tasking to meet safety and mission objectives	- Extensive ground training in geology in lunar-like environments (e.g., Iceland) - Cue cards and cuff checklists - Geological callouts should be scientifically precise and succinct; excessive communication can crowd voice loops from other team members	- Executive function - Working memory - Semantic memory - Speech - Language
Off-nominal and emergency scenarios	- Difficult to effectively simulate in ground training - Stressful and unexpected in real-life	- Cuff checklists can be referred to for certain emergency procedures (e.g., ICR) - Effective communication with MCC is key	- Executive function - Cognitive flexibility - Working memory
5-12 s lunar (voice) communication delay	- Delay can lead to talking over each other, frustration, miscommunication	- Procedure words (e.g., “over”) used consistently in ground training - Extensive training with crewmembers to learn communication style and anticipate speech cadence	- Executive function - Cognitive flexibility - Speech - Language - Working memory

## Discussion

Ensuring safe decision-making on the Moon and Mars during future surface exploration EVA will require adequate characterization of the cognitive performance risks prior to, during, and following EVA. To be useful, this characterization should inform surface EVA concept of operations (ConOps), NASA standards and guidelines for medical operations in future Artemis missions, training prior to flight in simulation environments such as the NBL, just-in-time training in-flight, and countermeasures targeting all mission phases. The results of this CTA will assist in current efforts to identify the key cognitive domains for safe decision-making during surface EVA. Specifically, our results provide the foundation for three specific outcomes: (1) prioritized and targeted cognitive performance measurement and functional performance tests, (2) EVA simulation design at varying levels of cognitive workload, and (3) the development of training and other technologies that can inform NASA standards and guidelines for medical operations and EVA planning in future Artemis missions to the Moon and Mars. We discuss the implications of our research for each of the three outcomes below.

The CTA can inform functional cognitive and performance testing related to the identified cognitive domains and provide a link between cognitive domains and operationally relevant performance metrics that can be tracked by NASA mission operations to ensure safe decision-making for exploration class mission EVA. For example, a cognitive domain that has previously shown decrements in spaceflight and EVA settings is vigilant attention^4–6,29^. We identified vigilant attention as a cognitive domain underlying some of the most cognitively demanding surface exploration EVA tasks, including traverse, maintenance, and sample collection. This suggests value to incorporating widely used tests of vigilant or sustained attention, such as the psychomotor vigilance test^30^, into EVA operations and research settings.

One of the most immediate utilities of this CTA is to inform current and future EVA simulation research. As NASA prepares to return to the Moon and eventually explore Mars, there is a need to understand which surface EVA tasks are most cognitively demanding and the key knowledge, skills, and abilities that are required for effective performance of those tasks. In addition to the qualitative data gained from our interviews, the quantitative cognitive demand ratings can be used to inform future surface EVA simulation study design and NASA standards and guidelines regarding cognitive workload. For example, the astronauts in our study highlighted EVA Prep/Post Ops as a particularly cognitively demanding aspect of EVA, yet many current EVA simulations omit this EVA task. In addition, the cognitive demand ratings of surface EVA tasks made by experts in this CTA could be compared to NASA-TLX ratings collected in other studies with EVA-like tasks. Such a comparison could most optimally inform EVA planning and scheduling on future exploration missions.

Findings on the specific cognitive demands that astronauts will face during surface exploration EVA can aid in the creation of products for cognitive performance monitoring capabilities and technologies, such as real-time data dashboards, that can be used by team members to keep track of cognitive workload and performance and automatically adjust EVA timelines as needed to manage cognitive workload. The development of embedded and unobtrusive cognitive measures can inform planning models and feed into dashboards that can be monitored by MCC. However, further research into what information should be conveyed to crew (i.e., what is useful as feedback without adding intensively to psychological demands), and how such technology could specifically aid future Mars missions, is needed. Given that the astronauts in our study rated the surface EVA tasks as overall less cognitively demanding than EVA operations and EVA research experts, it is important to understand the optimal types and amount of data that should be presented in a cognitive monitoring dashboard to future astronauts, and how viewing this data may influence astronaut stress, mood, and performance.

The information gathered in this CTA suggests several high-value research targets and recommendations for future exploration class mission EVA. Recommendations fall into several broad categories. The first recommendation is the importance of cognitive workload in off-nominal or contingency scenarios. Crew pre-mission training should include off-nominal and emergency scenarios such as ICR to prepare for low-likelihood but cognitively demanding and high consequence events. Physical workload and pain should be recognized as factors that can impact cognitive workload and managed accordingly prior to, during, and following EVA.

The second recommendation regards the factors that need to be included in EVA research and training. Situational awareness (SA) needs to be systematically measured in EVA research to characterize how and when there are decrements in SA that could affect performance. Factors that might add to cognitive workload experienced during surface EVA include inadequate sleep and circadian misalignment, which may be more likely with new vehicles and multiple complex mission phases^27^). On the lunar surface, astronauts will need as much navigational aid as technologically possible. To keep communication effective during lunar communication delays, effort should be made in EVA simulation and training to implement consistent procedure words (e.g., “over”, “copy”) to deal with the challenges of communication delay.

The third recommendation regards EVA planning and technology considerations. During lunar or Mars surface geological operations, job aids that reduce cognitive workload of difficult tasks should be incorporated. For example, cue cards on astronaut cuffs or a heads-up display (HUD) could include reminders of difficult geological terms to reduce cognitive workload associated with geology callouts. MCC should consider offloading some of the operator tasks of EVs while performing field geology to maximize the quality of science conducted on the surface. Crewmembers should be given extra time for suit mobility adaptation at the start of EVA operations given the cognitive workload associated with this EVA task. If extra time for suit adaptation is not built into the EVA timeline—separate from traverse given the cognitive workload associated with that EVA task—there is likely to be increased cognitive workload early in the EVA as crewmembers adapt to moving in the suit in partial gravity. Consideration of suit adaptation over the course of multiple mission phases is also critical.

The fourth recommendation notes that teamwork can be an effective countermeasure. To maximize the cognitive offloading of effective teamwork, crews should receive training to learn about cognitive offloading after being assigned to a particular mission and crew, in addition to training on managing different work styles, managing cognitive workload through teamwork, and other aspects of effective teamwork for surface EVA, such as tips for optimizing communication with lunar or Mars communication delays.

There are several limitations to consider in the present research. First, the mixed-methods design of the study necessitated a relatively small sample of experts, with the primary goal of eliciting in-depth qualitative data. Constraints on scheduling and availability motivated the decision to limit interviews to 1-h in duration. Because interviews were semi-structured, not every expert interviewed covered all planned topics. Although we intentionally sought to interview experts of different levels of EVA experience and from different occupational backgrounds, and several steps were taken to reduce response bias, differences in background between experts may have contributed to bias in responses. Finally, although the astronauts interviewed in the present study were highly experienced overall, no currently active astronauts have yet completed a surface EVA, and so our CTA is best characterized as a future oriented CTA. Feedback or subsequent CTAs should be gathered from astronauts as they complete surface EVAs on the Moon to most optimally characterize the cognitive demands associated with surface EVA.

Future research is needed to understand the cognitive capabilities required for surface EVA in future Mars missions, which will have unique challenges with respect to communication delay, behavioral health and performance countermeasures, and crew autonomy compared to lunar missions. In the present study, we asked experts how a lunar (voice) communication delay of 5-12 s might impact cognitive workload. It is unknown whether the concerns raised by experts for lunar communication delay, including frustration and miscommunication with potentially life-threatening consequences, scales linearly as the length of communication delay increases to Mars-like durations. It is likely that a different paradigm of ground-to-crew communication is needed at maximum Earth-to-Mars communication delay durations, which will require astronauts to conduct EVAs autonomously from Earth support. More research is needed to understand how both lunar and Mars-like communication delays impact astronaut and ground crew interactions, performance, and teamwork on EVAs. Finally, as NASA prepares to return to the Moon and eventually to Mars, it will be important to continue to learn from and leverage the valuable experiences of Apollo-era astronauts, such as in the Apollo Medical Operations Project^8^. Many current astronauts are also gaining valuable experience conducting simulated surface EVA on Earth, such as in NASA’s Joint EVA & Human Surface Mobility Test Team (JETT)^31^. Future research should continue to leverage the crucial experience from astronauts past and present to fully prepare the astronauts of the future for successful missions to the Moon and Mars.

## Supplementary information

CTA_Manuscript_Revision_SupplementalMaterials.

## Data Availability

The datasets used and/or analyzed during the current study are available from the corresponding author or by contacting NASA’s Behavioral Health and Performance Laboratory on reasonable request.

## References

[1] Chappell, S. P. et al. Evidence report: risk of injury and compromised performance due to EVA operations. (U.S. National Aeronautics and Space Administration (NASA). 2017).

[2] Strangman, G. E., Sipes, W. & Beven, G. Human cognitive performance in spaceflight and analogue environments. *Aviat. Space Environ. Med.* **85**, 1033–1048 (2014). 25245904 10.3357/ASEM.3961.2014

[3] Clément, G. R. et al. Challenges to the central nervous system during human spaceflight missions to Mars. *J. Neurophysiol.* **123**, 2037–2063 (2020). 32292116 10.1152/jn.00476.2019

[4] Dev, S. I. et al. Cognitive performance in ISS astronauts on 6-month low earth orbit missions. *Front. Physiol.* **15**, 1451269 (2024). 39633651 10.3389/fphys.2024.1451269 PMC11614644

[5] Tu, D. et al. Dynamic ensemble prediction of cognitive performance in spaceflight. *Sci. Rep.* **12**, 11032 (2022). 35773291 10.1038/s41598-022-14456-8 PMC9246897

[6] Nasrini, J. et al. Cognitive performance during confinement and sleep restriction in NASA’s human exploration research analog (HERA). *Front. Physiol.* **11**, 394 (2020). 32411017 10.3389/fphys.2020.00394 PMC7198903

[7] Garrett-Bakelman, F. E. et al. The NASA twins study: a multidimensional analysis of a year-long human spaceflight. *Science* **364**, eaau8650 (2019). 30975860 10.1126/science.aau8650 PMC7580864

[8] Scheuring, R. A. et al. The Apollo Medical Operations Project: Recommendations to improve crew health and performance for future exploration missions and lunar surface operations. *Acta Astronaut* **63**, 980–987 (2008).

[9] Rai, B. & Kaur, J. Mental and physical workload, salivary stress biomarkers and taste perception: Mars desert research station expedition. *N. Am. J. Med. Sci.* **4**, 577 (2012). 23181230 10.4103/1947-2714.103318 PMC3503377

[10] Smith, C. M., Segovia, M. D. & Salmon, O. F. Impact of reduced weight on motor and cognitive function in astronaut analogs: a simulated lunar gravity workload study. *Acta Astronaut* **206**, 18–29 (2023).

[11] Möller, F., Hoffmann, U., Vogt, T. & Steinberg, F. Exercise-related effects on executive functions during a simulated underwater extravehicular activity. *Hum. Factors* **65**, 1014–1028 (2023). 34340575 10.1177/00187208211032868

[12] Schlotman, T. E. et al. A Preliminary Assessment of Cognition and Fatigue During Simulated Lunar Surface Extravehicular Activities. In *AsMA 93rd Annual Aerospace Medicine Scientific Meeting*(New Orleans, LA, United States, 2023).

[13] Militello, L. G. & Hutton, R. J. Applied cognitive task analysis (ACTA): a practitioner’s toolkit for understanding cognitive task demands. *Ergonomics* **41**, 1618–1641 (1998). 9819578 10.1080/001401398186108

[14] Munson, B. & Holden, K. *Exploration Mission Tasks: A Technical Manual (NASA/TM-20210017320)*. (National Aeronauticsand Space Administration, Johnson Space Center Houston, Texas, 2021).

[15] Stuster, J., Adolf, J., Byrne, V. & Greene, M. *Generalizable Skills And Knowledge For Exploration Missions (NASA/CR-2018-22045).* (National Aeronautics and Space Administration, Johnson Space Center Houston, Texas, 2019).

[16] Hart, S. G. & Staveland, L. E. In *Advances in Psychology* Vol. 52, 139–183 (Elsevier, 1988).

[17] Walton, M. et al. Extravehicular activity on the lunar surface: Mapping mitigation risk consequence for crew needing assistance or rescue. *J. Space Saf. Eng.* **11**, 174–180 (2024).

[18] Ziemak, J. P., Dugan, B. A. & Rigby, C. K. *First-Level Supervisors Job Analysis Follow-Up: Identification**of KSAO-Task Linkages* (HUMAN RESOURCES RESEARCH ORGANIZATION ALEXANDRIA VA, 1994).

[19] Ionescu, T. Exploring the nature of cognitive flexibility. *N. Ideas Psychol.* **30**, 190–200 (2012).

[20] Nowrangi, M. A., Lyketsos, C., Rao, V. & Munro, C. A. Systematic review of neuroimaging correlates of executive functioning: converging evidence from different clinical populations. *J. Neuropsychiatry Clin. Neurosci.* **26**, 114–125 (2014). 24763759 10.1176/appi.neuropsych.12070176 PMC5171230

[21] Uddin, L. Q. Cognitive and behavioural flexibility: neural mechanisms and clinical considerations. *Nat. Rev. Neurosci.* **22**, 167–179 (2021). 33536614 10.1038/s41583-021-00428-w PMC7856857

[22] Langner, R. & Eickhoff, S. B. Sustaining attention to simple tasks: a meta-analytic review of the neural mechanisms of vigilant attention. *Psychol. Bull.* **139**, 870 (2013). 23163491 10.1037/a0030694 PMC3627747

[23] Kravitz, D. J., Saleem, K. S., Baker, C. I. & Mishkin, M. A new neural framework for visuospatial processing. *Nat. Rev. Neurosci.* **12**, 217–230 (2011). 21415848 10.1038/nrn3008 PMC3388718

[24] Emch, M., Von Bastian, C. C. & Koch, K. Neural correlates of verbal working memory: an fMRI meta-analysis. *Front. Hum. Neurosci.* **13**, 180 (2019). 31244625 10.3389/fnhum.2019.00180 PMC6581736

[25] Bookheimer, S. Functional MRI of language: new approaches to understanding the cortical organization of semantic processing. *Annu. Rev. Neurosci.* **25**, 151–188 (2002). 12052907 10.1146/annurev.neuro.25.112701.142946

[26] Paulin, M. G. The role of the cerebellum in motor control and perception. *Brain Behav. Evolut.* **41**, 39–50 (1993). 10.1159/000113822 8431754

[27] Flynn-Evans, E. E., Barger, L. K., Kubey, A. A., Sullivan, J. P. & Czeisler, C. A. Circadian misalignment affects sleep and medication use before and during spaceflight. *npj Microgravity* **2**, 1–6 (2016). 28725719 10.1038/npjmgrav.2015.19 PMC5515517

[28] Dolcos, F., Iordan, A. D. & Dolcos, S. Neural correlates of emotion–cognition interactions: a review of evidence from brain imaging investigations. *J. Cogn. Psychol.* **23**, 669–694 (2011). 10.1080/20445911.2011.594433 PMC3206704 22059115

[29] Schlotman, T. E. et al. In *IEEE Aerospace Conference* 1–10 (IEEE, 2023).

[30] Basner, M. & Dinges, D. F. Maximizing sensitivity of the psychomotor vigilance test (PVT) to sleep loss. *Sleep* **34**, 581–591 (2011). 21532951 10.1093/sleep/34.5.581 PMC3079937

[31] Caswell, T. et al. In *54th Lunar and Planetary Science Conference*.

