# Supplementary material for: Identifying cognitive capabilities required for optimal surface extravehicular activity performance
Source: NPJ Microgravity. 2025 Dec 6;12:4. doi: 10.1038/s41526-025-00545-1 (PMC12775082; doi:10.1038/s41526-025-00545-1)
Supplement: Supplementary file 1 — CTA_Manuscript_Revision_SupplementalMaterials. [file 41526_2025_545_MOESM1_ESM.docx]

**Supplemental Table 1**

*Expert (n = 15) Cognitive Demand Ratings of EVA Tasks*

| **Surface EVA Task** | **Cognitive Demand Rating Mean (SD)** |
| --- | --- |
| EVA Prep/Post Ops | 61.0 (30.9) |
| Inspection of Surroundings | 52.6 (25.3) |
| Sample Identification | 51.8 (26.3) |
| Sample Collection Rock | 47.0 (23.5) |
| Sample Collection Regolith | 57.2 (25.5) |
| Surface Mobility | 58.4 (26.3) |
| Maintenance Tasks | 58.0 (20.9) |
| Payload Operations | 54.1 (25.2) |
| Public Affairs Operations | 39.3 (23.6) |
| Traverse | 72.4 (24.2) |
| Worksite setup cleanup | 45.6 (16.0) |

**Supplemental Table 2**

*Expert (n = 9) Cognitive Demand Ratings of EVA Subtasks*

|  | **Cognitive Demand Rating** |
| --- | --- |
| **Ambulation (after adaptation) (N=1)** | Mean (SD): 70.0 (NA) Median [Min, Max]: 70.0 [70.0, 70.0] |
| **Bulk Scoop sample (N=7)** | Mean (SD): 42.1 (22.7) Median [Min, Max]: 50.0 [10.0, 60.0] |
| **Cable Management (Burying) (N=7)** | Mean (SD): 54.3 (19.7) Median [Min, Max]: 60.0 [20.0, 80.0] |
| **Cable Management (Routing) (N=8)** | Mean (SD): 56.9 (17.1) Median [Min, Max]: 62.5 [20.0, 70.0] |
| **Chip sample (N=7)** | Mean (SD): 65.0 (25.5) Median [Min, Max]: 75.0 [20.0, 90.0] |
| **Core sample (double) (N=6)** | Mean (SD): 73.3 (32.8) Median [Min, Max]: 82.5 [10.0, 100] |
| **Core sample (single) (N=7)** | Mean (SD): 63.6 (34.0) Median [Min, Max]: 80.0 [10.0, 90.0] |
| **Deploy Retrieve Payload (N=7)** | Mean (SD): 63.9 (23.1) Median [Min, Max]: 65.0 [17.0, 85.0] |
| **Depressurization/repressurization (N=1)** | Mean (SD): 90.0 (NA) Median [Min, Max]: 90.0 [90.0, 90.0] |
| **Descend Ascend Ladder (N=7)** | Mean (SD): 59.3 (27.1) Median [Min, Max]: 60.0 [20.0, 95.0] |
| **Descend Ascend Steps (N=7)** | Mean (SD): 57.9 (27.4) Median [Min, Max]: 60.0 [20.0, 90.0] |
| **Determine sample priority (N=1)** | Mean (SD): 60.0 (NA) Median [Min, Max]: 60.0 [60.0, 60.0] |
| **Determine sample representativeness of site (N=1)** | Mean (SD): 75.0 (NA) Median [Min, Max]: 75.0 [75.0, 75.0] |
| **Don Doff Suit (N=8)** | Mean (SD): 56.3 (30.1) Median [Min, Max]: 60.0 [20.0, 90.0] |
| **Don Doff Suit (support IV) (N=1)** | Mean (SD): 80.0 (NA) Median [Min, Max]: 80.0 [80.0, 80.0] |
| **Dust Mitigation (Clean Equipment) (N=7)** | Mean (SD): 45.0 (31.1) Median [Min, Max]: 25.0 [20.0, 95.0] |
| **EVA Adaptation (ambulation) (N=7)** | Mean (SD): 53.6 (20.1) Median [Min, Max]: 50.0 [20.0, 80.0] |
| **EVA Adaptation (postural change kneeling) (N=7)** | Mean (SD): 63.6 (19.5) Median [Min, Max]: 70.0 [30.0, 85.0] |
| **Flag Deployment (N=8)** | Mean (SD): 39.4 (26.4) Median [Min, Max]: 42.5 [0, 80.0] |
| **Float sample (N=7)** | Mean (SD): 51.4 (24.1) Median [Min, Max]: 60.0 [10.0, 80.0] |
| **Geological Description (N=3)** | Mean (SD): 88.3 (2.89) Median [Min, Max]: 90.0 [85.0, 90.0] |
| **Identify sample (N=3)** | Mean (SD): 60.0 (18.0) Median [Min, Max]: 65.0 [40.0, 75.0] |
| **Inspection (N=9)** | Mean (SD): 60.0 (27.4) Median [Min, Max]: 65.0 [20.0, 100] |
| **Mating Demating Connectors (N=8)** | Mean (SD): 57.5 (18.3) Median [Min, Max]: 60.0 [20.0, 80.0] |
| **Navigation (N=1)** | Mean (SD): 75.0 (NA) Median [Min, Max]: 75.0 [75.0, 75.0] |
| **Open Close Hatch (N=8)** | Mean (SD): 61.9 (29.1) Median [Min, Max]: 72.5 [15.0, 90.0] |
| **Open Close Hatch (First Time) (N=1)** | Mean (SD): 70.0 (NA) Median [Min, Max]: 70.0 [70.0, 70.0] |
| **Open Close Hatch (Subsequent Times) (N=1)** | Mean (SD): 40.0 (NA) Median [Min, Max]: 40.0 [40.0, 40.0] |
| **Photography (N=9)** | Mean (SD): 41.1 (21.6) Median [Min, Max]: 35.0 [20.0, 80.0] |
| **Place Sample Marker (N=7)** | Mean (SD): 47.1 (25.1) Median [Min, Max]: 50.0 [10.0, 75.0] |
| **Plaque Deployment (N=8)** | Mean (SD): 30.6 (22.9) Median [Min, Max]: 30.0 [0, 65.0] |
| **Postural change (after adaptation) (N=1)** | Mean (SD): 75.0 (NA) Median [Min, Max]: 75.0 [75.0, 75.0] |
| **Prebreathe (N=2)** | Mean (SD): 90.0 (0) Median [Min, Max]: 90.0 [90.0, 90.0] |
| **Public Speaking (N=1)** | Mean (SD): 80.0 (NA) Median [Min, Max]: 80.0 [80.0, 80.0] |
| **Rake sample (N=7)** | Mean (SD): 47.9 (23.4) Median [Min, Max]: 60.0 [10.0, 70.0] |
| **Removing Installing (Fasteners) (N=8)** | Mean (SD): 58.1 (16.9) Median [Min, Max]: 62.5 [20.0, 70.0] |
| **Removing Installing (Hardware) (N=8)** | Mean (SD): 55.0 (18.3) Median [Min, Max]: 60.0 [20.0, 80.0] |
| **Rover Driving (N=1)** | Mean (SD): 80.0 (NA) Median [Min, Max]: 80.0 [80.0, 80.0] |
| **Sample retrieval (N=8)** | Mean (SD): 48.1 (16.7) Median [Min, Max]: 50.0 [20.0, 75.0] |
| **Storage (N=8)** | Mean (SD): 46.9 (16.9) Median [Min, Max]: 50.0 [15.0, 75.0] |
| **Suit checks and procedures (solo) (N=2)** | Mean (SD): 92.5 (3.54) Median [Min, Max]: 92.5 [90.0, 95.0] |
| **Suit checks and procedures (with good help) (N=1)** | Mean (SD): 70.0 (NA) Median [Min, Max]: 70.0 [70.0, 70.0] |
| **Surface collection sample (N=7)** | Mean (SD): 46.4 (22.5) Median [Min, Max]: 55.0 [10.0, 70.0] |
| **Tool configuration (N=1)** | Mean (SD): 80.0 (NA) Median [Min, Max]: 80.0 [80.0, 80.0] |
| **Tool sample retrieval (N=8)** | Mean (SD): 41.9 (16.0) Median [Min, Max]: 50.0 [20.0, 60.0] |
| **Tracking the timeline (N=1)** | Mean (SD): 10.0 (NA) Median [Min, Max]: 10.0 [10.0, 10.0] |
| **Transport Payload (with cart) (N=7)** | Mean (SD): 50.0 (24.3) Median [Min, Max]: 50.0 [20.0, 75.0] |
| **Transport Payload (without cart) (N=7)** | Mean (SD): 48.6 (28.7) Median [Min, Max]: 50.0 [15.0, 80.0] |
| **Traverse (in darkness) (N=1)** | Mean (SD): 90.0 (NA) Median [Min, Max]: 90.0 [90.0, 90.0] |
| **Traverse (in sun) (N=1)** | Mean (SD): 70.0 (NA) Median [Min, Max]: 70.0 [70.0, 70.0] |
| **Traverse with cart (N=1)** | Mean (SD): 85.0 (NA) Median [Min, Max]: 85.0 [85.0, 85.0] |
| **Traverse without cart (N=8)** | Mean (SD): 49.4 (24.8) Median [Min, Max]: 45.0 [15.0, 90.0] |
| **Traverse without cart (without navigation) (N=1)** | Mean (SD): 85.0 (NA) Median [Min, Max]: 85.0 [85.0, 85.0] |
| **Trench sample (N=7)** | Mean (SD): 56.4 (25.6) Median [Min, Max]: 70.0 [20.0, 80.0] |
| **Understand location (N=2)** | Mean (SD): 77.5 (3.54) Median [Min, Max]: 77.5 [75.0, 80.0] |

**Supplemental Table 3**

*Expert (n = 6) Cognitive Demand Ratings of Knowledge, Skills, and Abilities (KSAs)*

|  | **Cognitive Demand Rating** |
| --- | --- |
| **Ability to apply knowledge of consumables rate to adjust your own body (N=1)** | Mean (SD): 90.0 (NA) Median [Min, Max]: 90.0 [90.0, 90.0] Missing: 0 (0%) |
| **Ability to deploy sample marker based on identification (N=1)** | Mean (SD): 40.0 (NA) Median [Min, Max]: 40.0 [40.0, 40.0] Missing: 0 (0%) |
| **Ability to document terrain with photo documentation of terrain features to MCC (N=1)** | Mean (SD): 30.0 (NA) Median [Min, Max]: 30.0 [30.0, 30.0] Missing: 0 (0%) |
| **Ability to orient yourself to where you are on the surface (referring to maps) (N=1)** | Mean (SD): 40.0 (NA) Median [Min, Max]: 40.0 [40.0, 40.0] Missing: 0 (0%) |
| **Ability to pay attention and follow the mission timeline, keeping track of where you are on the timeline, where you are supposed to be (N=1)** | Mean (SD): 60.0 (NA) Median [Min, Max]: 60.0 [60.0, 60.0] Missing: 0 (0%) |
| **Ability to provide a structured and helpful description of what is being seen (being able to describe terrain features like craters, plains at different scales) (N=1)** | Mean (SD): 60.0 (NA) Median [Min, Max]: 60.0 [60.0, 60.0] Missing: 0 (0%) |
| **Accurately communicate what you are seeing and experiencing (N=1)** | Mean (SD): 60.0 (NA) Median [Min, Max]: 60.0 [60.0, 60.0] Missing: 0 (0%) |
| **Act of sampling (N=1)** | Mean (SD): 40.0 (NA) Median [Min, Max]: 40.0 [40.0, 40.0] Missing: 0 (0%) |
| **Being able to ambulate (kneeling and bending) (N=1)** | Mean (SD): 80.0 (NA) Median [Min, Max]: 80.0 [80.0, 80.0] Missing: 0 (0%) |
| **Being able to manage secondary tasks (navigation, monitoring consumables) and prioritize tasks (N=1)** | Mean (SD): 90.0 (NA) Median [Min, Max]: 90.0 [90.0, 90.0] Missing: 0 (0%) |
| **Bringing the right equipment (the correct tools and items to execute maintenance tasks), pre-planning (N=1)** | Mean (SD): 80.0 (NA) Median [Min, Max]: 80.0 [80.0, 80.0] Missing: 0 (0%) |
| **Camping experience (N=1)** | Mean (SD): NA (NA) Median [Min, Max]: NA [NA, NA] Missing: 1 (100%) |
| **Choosing the right tool for sampling (N=1)** | Mean (SD): 20.0 (NA) Median [Min, Max]: 20.0 [20.0, 20.0] Missing: 0 (0%) |
| **Communicate with geology terminology to ground (N=2)** | Mean (SD): 80.0 (0) Median [Min, Max]: 80.0 [80.0, 80.0] Missing: 0 (0%) |
| **Communicating all of the details of your finding back to MCC (N=1)** | Mean (SD): 20.0 (NA) Median [Min, Max]: 20.0 [20.0, 20.0] Missing: 0 (0%) |
| **Communicating with MCC on the procedures (N=1)** | Mean (SD): 15.0 (NA) Median [Min, Max]: 15.0 [15.0, 15.0] Missing: 0 (0%) |
| **Communicating with other EV to not duplicate effort (N=1)** | Mean (SD): 90.0 (NA) Median [Min, Max]: 90.0 [90.0, 90.0] Missing: 0 (0%) |
| **Communication back to MCC of what you’re doing (N=1)** | Mean (SD): 20.0 (NA) Median [Min, Max]: 20.0 [20.0, 20.0] Missing: 0 (0%) |
| **Communication with EV buddy, IV, MCC (N=1)** | Mean (SD): 10.0 (NA) Median [Min, Max]: 10.0 [10.0, 10.0] Missing: 0 (0%) |
| **Consumables rates (N=1)** | Mean (SD): 95.0 (NA) Median [Min, Max]: 95.0 [95.0, 95.0] Missing: 0 (0%) |
| **Describe (from macro to micro descriptions, from hills in distance to rocks at feet) (N=1)** | Mean (SD): 70.0 (NA) Median [Min, Max]: 70.0 [70.0, 70.0] Missing: 0 (0%) |
| **Describe samples (N=1)** | Mean (SD): 70.0 (NA) Median [Min, Max]: 70.0 [70.0, 70.0] Missing: 0 (0%) |
| **Describe the colors of sample (N=1)** | Mean (SD): 20.0 (NA) Median [Min, Max]: 20.0 [20.0, 20.0] Missing: 0 (0%) |
| **Describe the major components of sample (minerals, etc.) (N=1)** | Mean (SD): 60.0 (NA) Median [Min, Max]: 60.0 [60.0, 60.0] Missing: 0 (0%) |
| **Describe the size and shape of sample (N=1)** | Mean (SD): 20.0 (NA) Median [Min, Max]: 20.0 [20.0, 20.0] Missing: 0 (0%) |
| **Describe the texture of sample (N=1)** | Mean (SD): 40.0 (NA) Median [Min, Max]: 40.0 [40.0, 40.0] Missing: 0 (0%) |
| **Describing geology (N=1)** | Mean (SD): 90.0 (NA) Median [Min, Max]: 90.0 [90.0, 90.0] Missing: 0 (0%) |
| **Describing the sample in the bag (N=1)** | Mean (SD): 70.0 (NA) Median [Min, Max]: 70.0 [70.0, 70.0] Missing: 0 (0%) |
| **Determine which rock to pick up (N=1)** | Mean (SD): 50.0 (NA) Median [Min, Max]: 50.0 [50.0, 50.0] Missing: 0 (0%) |
| **Document (photographs and other 360 panoramas) (N=1)** | Mean (SD): 40.0 (NA) Median [Min, Max]: 40.0 [40.0, 40.0] Missing: 0 (0%) |
| **Documenting the sample by taking pictures of its location relative to other features (using object for scale) (N=1)** | Mean (SD): 30.0 (NA) Median [Min, Max]: 30.0 [30.0, 30.0] Missing: 0 (0%) |
| **Documenting with video and camera in the moment (N=1)** | Mean (SD): 50.0 (NA) Median [Min, Max]: 50.0 [50.0, 50.0] Missing: 0 (0%) |
| **Don Doff Suit (N=1)** | Mean (SD): 80.0 (NA) Median [Min, Max]: 80.0 [80.0, 80.0] Missing: 0 (0%) |
| **Drive tube procedures (N=1)** | Mean (SD): 70.0 (NA) Median [Min, Max]: 70.0 [70.0, 70.0] Missing: 0 (0%) |
| **Dust mitigation at doffing of suit (N=1)** | Mean (SD): 10.0 (NA) Median [Min, Max]: 10.0 [10.0, 10.0] Missing: 0 (0%) |
| **Experience practicing ambulation in ARGOS/NBL (N=1)** | Mean (SD): 80.0 (NA) Median [Min, Max]: 80.0 [80.0, 80.0] Missing: 0 (0%) |
| **Familiarity with different types of geological formations (N=1)** | Mean (SD): 90.0 (NA) Median [Min, Max]: 90.0 [90.0, 90.0] Missing: 0 (0%) |
| **Features of equipment (N=1)** | Mean (SD): 80.0 (NA) Median [Min, Max]: 80.0 [80.0, 80.0] Missing: 0 (0%) |
| **Following procedures for airlock operations (based on training) (N=1)** | Mean (SD): 20.0 (NA) Median [Min, Max]: 20.0 [20.0, 20.0] Missing: 0 (0%) |
| **Following procedures for donning/doffing suit (N=1)** | Mean (SD): 15.0 (NA) Median [Min, Max]: 15.0 [15.0, 15.0] Missing: 0 (0%) |
| **Following procedures in right order, not skipping any steps, making frequent suit checks (N=1)** | Mean (SD): 20.0 (NA) Median [Min, Max]: 20.0 [20.0, 20.0] Missing: 0 (0%) |
| **Geological knowledge (N=1)** | Mean (SD): 80.0 (NA) Median [Min, Max]: 80.0 [80.0, 80.0] Missing: 0 (0%) |
| **Geological terminology (N=1)** | Mean (SD): 80.0 (NA) Median [Min, Max]: 80.0 [80.0, 80.0] Missing: 0 (0%) |
| **Geology training on ground (N=1)** | Mean (SD): 50.0 (NA) Median [Min, Max]: 50.0 [50.0, 50.0] Missing: 0 (0%) |
| **Ground training (N=1)** | Mean (SD): 80.0 (NA) Median [Min, Max]: 80.0 [80.0, 80.0] Missing: 0 (0%) |
| **Ground training on core samples (N=1)** | Mean (SD): 40.0 (NA) Median [Min, Max]: 40.0 [40.0, 40.0] Missing: 0 (0%) |
| **Having fun and excitement (N=1)** | Mean (SD): NA (NA) Median [Min, Max]: NA [NA, NA] Missing: 1 (100%) |
| **How best to take the sample correctly in terms of body posture (in terms of injury, performance, metabolic cost, etc.) so as not to wear yourself out (N=1)** | Mean (SD): 20.0 (NA) Median [Min, Max]: 20.0 [20.0, 20.0] Missing: 0 (0%) |
| **How the equipment works (N=1)** | Mean (SD): 90.0 (NA) Median [Min, Max]: 90.0 [90.0, 90.0] Missing: 0 (0%) |
| **How to deploy the flag in low illumination (N=1)** | Mean (SD): 50.0 (NA) Median [Min, Max]: 50.0 [50.0, 50.0] Missing: 0 (0%) |
| **How to deploy the plaque in low illumination (N=1)** | Mean (SD): 50.0 (NA) Median [Min, Max]: 50.0 [50.0, 50.0] Missing: 0 (0%) |
| **How to do the maintenance for each type of equipment (N=1)** | Mean (SD): 90.0 (NA) Median [Min, Max]: 90.0 [90.0, 90.0] Missing: 0 (0%) |
| **Inspecting your equipment (N=1)** | Mean (SD): 80.0 (NA) Median [Min, Max]: 80.0 [80.0, 80.0] Missing: 0 (0%) |
| **Interactions of the different equipment (how everything connects) (N=1)** | Mean (SD): 80.0 (NA) Median [Min, Max]: 80.0 [80.0, 80.0] Missing: 0 (0%) |
| **Know geology terms (N=1)** | Mean (SD): 80.0 (NA) Median [Min, Max]: 80.0 [80.0, 80.0] Missing: 0 (0%) |
| **Know how to use tools correctly (N=1)** | Mean (SD): 40.0 (NA) Median [Min, Max]: 40.0 [40.0, 40.0] Missing: 0 (0%) |
| **Knowing how to ambulate in a really efficient manner (N=1)** | Mean (SD): 85.0 (NA) Median [Min, Max]: 85.0 [85.0, 85.0] Missing: 0 (0%) |
| **Knowing how to deploy sample marker (N=1)** | Mean (SD): 30.0 (NA) Median [Min, Max]: 30.0 [30.0, 30.0] Missing: 0 (0%) |
| **Knowing how to interact with your environment in the suit (N=1)** | Mean (SD): 80.0 (NA) Median [Min, Max]: 80.0 [80.0, 80.0] Missing: 0 (0%) |
| **Knowing how to use tools correctly (N=1)** | Mean (SD): 30.0 (NA) Median [Min, Max]: 30.0 [30.0, 30.0] Missing: 0 (0%) |
| **Knowing how to work within the suit's limitations (N=4)** | Mean (SD): 57.5 (43.5) Median [Min, Max]: 60.0 [15.0, 95.0] Missing: 0 (0%) |
| **Knowing the design and function of your equipment (N=1)** | Mean (SD): 80.0 (NA) Median [Min, Max]: 80.0 [80.0, 80.0] Missing: 0 (0%) |
| **Knowing the procedures of how to deploy flag and plaque (N=1)** | Mean (SD): 25.0 (NA) Median [Min, Max]: 25.0 [25.0, 25.0] Missing: 0 (0%) |
| **Knowing where to put the flag and plaque (N=1)** | Mean (SD): 25.0 (NA) Median [Min, Max]: 25.0 [25.0, 25.0] Missing: 0 (0%) |
| **Knowing your sampling capacity—how much sample is required? Are there defined objectives or constraints in terms of the rock/regolith samples that are required. (N=1)** | Mean (SD): 20.0 (NA) Median [Min, Max]: 20.0 [20.0, 20.0] Missing: 0 (0%) |
| **Knowledge and skills of how to correctly and adequately store the sample (get it in the container and seal it without contaminating it) (N=1)** | Mean (SD): 20.0 (NA) Median [Min, Max]: 20.0 [20.0, 20.0] Missing: 0 (0%) |
| **Knowledge of environmental and topographical constraints to suit mobility (slopes over a certain degree, sharp looking rocks) (N=1)** | Mean (SD): 30.0 (NA) Median [Min, Max]: 30.0 [30.0, 30.0] Missing: 0 (0%) |
| **Knowledge of EVA science objectives (N=1)** | Mean (SD): 15.0 (NA) Median [Min, Max]: 15.0 [15.0, 15.0] Missing: 0 (0%) |
| **Knowledge of how the suit articulates (pivots, how parts rotate; different from natural movement) (N=1)** | Mean (SD): 10.0 (NA) Median [Min, Max]: 10.0 [10.0, 10.0] Missing: 0 (0%) |
| **Knowledge of how to address off-nominal situations with tools (i.e., stuck drill)—more pertaining to regolith sample (N=1)** | Mean (SD): 70.0 (NA) Median [Min, Max]: 70.0 [70.0, 70.0] Missing: 0 (0%) |
| **Knowledge of how to avoid and/or recover from suboptimal positions (N=1)** | Mean (SD): 5.00 (NA) Median [Min, Max]: 5.00 [5.00, 5.00] Missing: 0 (0%) |
| **Knowledge of how to use geology tools correctly (N=1)** | Mean (SD): 20.0 (NA) Median [Min, Max]: 20.0 [20.0, 20.0] Missing: 0 (0%) |
| **Knowledge of how what you are seeing relates to what you have seen elsewhere at other sites on the Moon or what you have sampled elsewhere on the Moon during that mission (depends on recency) (N=1)** | Mean (SD): 50.0 (NA) Median [Min, Max]: 50.0 [50.0, 50.0] Missing: 0 (0%) |
| **Knowledge of off-nominal scenarios (vigilance for emergency) (N=1)** | Mean (SD): 15.0 (NA) Median [Min, Max]: 15.0 [15.0, 15.0] Missing: 0 (0%) |
| **Knowledge of specific sample site and how it relates to science objectives (N=1)** | Mean (SD): 40.0 (NA) Median [Min, Max]: 40.0 [40.0, 40.0] Missing: 0 (0%) |
| **Knowledge of the nominal procedures (more familiarity, less cognitive demand) (N=1)** | Mean (SD): 50.0 (NA) Median [Min, Max]: 50.0 [50.0, 50.0] Missing: 0 (0%) |
| **Knowledge of the way suit moves (N=1)** | Mean (SD): 40.0 (NA) Median [Min, Max]: 40.0 [40.0, 40.0] Missing: 0 (0%) |
| **Knowledge of what geological features you are looking for (mission objectives) (N=1)** | Mean (SD): 60.0 (NA) Median [Min, Max]: 60.0 [60.0, 60.0] Missing: 0 (0%) |
| **Knowledge of what route to be taking, what to take with me (N=1)** | Mean (SD): 70.0 (NA) Median [Min, Max]: 70.0 [70.0, 70.0] Missing: 0 (0%) |
| **Knowledge of what you’re looking for and if you’ve found it (do you have the right sample) (N=1)** | Mean (SD): 60.0 (NA) Median [Min, Max]: 60.0 [60.0, 60.0] Missing: 0 (0%) |
| **Knowledge on geology sampling skills (N=1)** | Mean (SD): 30.0 (NA) Median [Min, Max]: 30.0 [30.0, 30.0] Missing: 0 (0%) |
| **Landing site specific knowledge or training (more training, less cognitive demand) (N=1)** | Mean (SD): 60.0 (NA) Median [Min, Max]: 60.0 [60.0, 60.0] Missing: 0 (0%) |
| **Location determination (N=1)** | Mean (SD): 80.0 (NA) Median [Min, Max]: 80.0 [80.0, 80.0] Missing: 0 (0%) |
| **Look (navigation, know where you are at) (N=1)** | Mean (SD): 60.0 (NA) Median [Min, Max]: 60.0 [60.0, 60.0] Missing: 0 (0%) |
| **Lots of complicated steps in the double drive tube procedures, requires referring to procedures instead of in memory (N=1)** | Mean (SD): 75.0 (NA) Median [Min, Max]: 75.0 [75.0, 75.0] Missing: 0 (0%) |
| **Making sure you are coordinating with IV and MCC on placement of flag and plaque (N=1)** | Mean (SD): 60.0 (NA) Median [Min, Max]: 60.0 [60.0, 60.0] Missing: 0 (0%) |
| **Making sure you brought the flag and plaque (N=1)** | Mean (SD): 60.0 (NA) Median [Min, Max]: 60.0 [60.0, 60.0] Missing: 0 (0%) |
| **Making sure you have enough sample markers (N=1)** | Mean (SD): 90.0 (NA) Median [Min, Max]: 90.0 [90.0, 90.0] Missing: 0 (0%) |
| **Mission operations, briefing, lunar topography, hazard analysis (N=1)** | Mean (SD): 95.0 (NA) Median [Min, Max]: 95.0 [95.0, 95.0] Missing: 0 (0%) |
| **Multi-tasking (N=1)** | Mean (SD): 90.0 (NA) Median [Min, Max]: 90.0 [90.0, 90.0] Missing: 0 (0%) |
| **Navigating on the surface and relating location to maps (N=1)** | Mean (SD): 50.0 (NA) Median [Min, Max]: 50.0 [50.0, 50.0] Missing: 0 (0%) |
| **Navigation skills (N=1)** | Mean (SD): 95.0 (NA) Median [Min, Max]: 95.0 [95.0, 95.0] Missing: 0 (0%) |
| **Navigational capabilities (navigational tool usage) (N=1)** | Mean (SD): 90.0 (NA) Median [Min, Max]: 90.0 [90.0, 90.0] Missing: 0 (0%) |
| **Observations (~10 minute walk around to observe terrain) (N=1)** | Mean (SD): 50.0 (NA) Median [Min, Max]: 50.0 [50.0, 50.0] Missing: 0 (0%) |
| **Photography operations (N=1)** | Mean (SD): 20.0 (NA) Median [Min, Max]: 20.0 [20.0, 20.0] Missing: 0 (0%) |
| **Physical ability (N=1)** | Mean (SD): NA (NA) Median [Min, Max]: NA [NA, NA] Missing: 1 (100%) |
| **Physical ability to don and doff suit (doffing harder than donning), assuming no help (N=1)** | Mean (SD): 80.0 (NA) Median [Min, Max]: 80.0 [80.0, 80.0] Missing: 0 (0%) |
| **Physical ability to hammer the double drive tube (N=1)** | Mean (SD): 60.0 (NA) Median [Min, Max]: 60.0 [60.0, 60.0] Missing: 0 (0%) |
| **Physical ability to pull out the double drive tube (N=1)** | Mean (SD): 60.0 (NA) Median [Min, Max]: 60.0 [60.0, 60.0] Missing: 0 (0%) |
| **Pick up the rock (N=1)** | Mean (SD): 50.0 (NA) Median [Min, Max]: 50.0 [50.0, 50.0] Missing: 0 (0%) |
| **Post-mission maintenance on suit, check for wear and tear (N=1)** | Mean (SD): 85.0 (NA) Median [Min, Max]: 85.0 [85.0, 85.0] Missing: 0 (0%) |
| **Properly labeling and documenting the sample (N=1)** | Mean (SD): 15.0 (NA) Median [Min, Max]: 15.0 [15.0, 15.0] Missing: 0 (0%) |
| **Provide interpretation of sample (N=1)** | Mean (SD): 50.0 (NA) Median [Min, Max]: 50.0 [50.0, 50.0] Missing: 0 (0%) |
| **Provide other distinguishing characteristics (N=1)** | Mean (SD): 20.0 (NA) Median [Min, Max]: 20.0 [20.0, 20.0] Missing: 0 (0%) |
| **Public speaking and communicating the big picture of what it is we’re doing and why it’s important to humanity (N=1)** | Mean (SD): 70.0 (NA) Median [Min, Max]: 70.0 [70.0, 70.0] Missing: 0 (0%) |
| **Retrieval of sample marker and tools (N=1)** | Mean (SD): 20.0 (NA) Median [Min, Max]: 20.0 [20.0, 20.0] Missing: 0 (0%) |
| **Sealing sample in the drive tube containers (N=1)** | Mean (SD): 70.0 (NA) Median [Min, Max]: 70.0 [70.0, 70.0] Missing: 0 (0%) |
| **Skill of using geology tools to take samples (N=1)** | Mean (SD): 25.0 (NA) Median [Min, Max]: 25.0 [25.0, 25.0] Missing: 0 (0%) |
| **Skill to communicate with general audience (public affairs operations) on why it’s important and why you are doing it (N=1)** | Mean (SD): 20.0 (NA) Median [Min, Max]: 20.0 [20.0, 20.0] Missing: 0 (0%) |
| **Skill to communicate with MCC on the technical nuts and bolts of what you are doing (N=1)** | Mean (SD): 20.0 (NA) Median [Min, Max]: 20.0 [20.0, 20.0] Missing: 0 (0%) |
| **Skill to deploy the flag and plaque (N=1)** | Mean (SD): 15.0 (NA) Median [Min, Max]: 15.0 [15.0, 15.0] Missing: 0 (0%) |
| **Skill to place the sample marker (N=1)** | Mean (SD): 15.0 (NA) Median [Min, Max]: 15.0 [15.0, 15.0] Missing: 0 (0%) |
| **Skill to tether/restrain to HLS (to be safe while high up) (N=1)** | Mean (SD): 30.0 (NA) Median [Min, Max]: 30.0 [30.0, 30.0] Missing: 0 (0%) |
| **Skill to use the geology tools correctly (N=1)** | Mean (SD): 40.0 (NA) Median [Min, Max]: 40.0 [40.0, 40.0] Missing: 0 (0%) |
| **Skill to use your camera to document environment so everyone else can see what you are looking at (N=1)** | Mean (SD): 20.0 (NA) Median [Min, Max]: 20.0 [20.0, 20.0] Missing: 0 (0%) |
| **Spatial orientation (where you are in relation to lunar features like carters, shadowed regions) (N=1)** | Mean (SD): 80.0 (NA) Median [Min, Max]: 80.0 [80.0, 80.0] Missing: 0 (0%) |
| **Stowing the sample (N=1)** | Mean (SD): 20.0 (NA) Median [Min, Max]: 20.0 [20.0, 20.0] Missing: 0 (0%) |
| **Suit and hatch emergency operations knowledge (N=1)** | Mean (SD): 100 (NA) Median [Min, Max]: 100 [100, 100] Missing: 0 (0%) |
| **Suit familiarity (N=1)** | Mean (SD): 80.0 (NA) Median [Min, Max]: 80.0 [80.0, 80.0] Missing: 0 (0%) |
| **Suit fit knowledge (N=1)** | Mean (SD): NA (NA) Median [Min, Max]: NA [NA, NA] Missing: 1 (100%) |
| **Suit operations knowledge (N=1)** | Mean (SD): 85.0 (NA) Median [Min, Max]: 85.0 [85.0, 85.0] Missing: 0 (0%) |
| **Take series of photos at specific distances and angles of sample (N=1)** | Mean (SD): 60.0 (NA) Median [Min, Max]: 60.0 [60.0, 60.0] Missing: 0 (0%) |
| **Taking pictures of samples (N=1)** | Mean (SD): 20.0 (NA) Median [Min, Max]: 20.0 [20.0, 20.0] Missing: 0 (0%) |
| **Taking the post-sample photos (N=1)** | Mean (SD): 40.0 (NA) Median [Min, Max]: 40.0 [40.0, 40.0] Missing: 0 (0%) |
| **Terminology for geological features (N=1)** | Mean (SD): 80.0 (NA) Median [Min, Max]: 80.0 [80.0, 80.0] Missing: 0 (0%) |
| **Tool management (N=1)** | Mean (SD): 50.0 (NA) Median [Min, Max]: 50.0 [50.0, 50.0] Missing: 0 (0%) |
| **Training on ground (N=1)** | Mean (SD): 40.0 (NA) Median [Min, Max]: 40.0 [40.0, 40.0] Missing: 0 (0%) |
| **Trenching based on illumination conditions (N=1)** | Mean (SD): 50.0 (NA) Median [Min, Max]: 50.0 [50.0, 50.0] Missing: 0 (0%) |
| **Understanding how low illumination impacts ability to identify correct samples (N=1)** | Mean (SD): 75.0 (NA) Median [Min, Max]: 75.0 [75.0, 75.0] Missing: 0 (0%) |
| **Understanding of how to get back (contingency return path); not exceeding return capability (consumables rate, dynamically changing) (N=1)** | Mean (SD): 80.0 (NA) Median [Min, Max]: 80.0 [80.0, 80.0] Missing: 0 (0%) |
| **Understanding science objectives; can get reminders from MCC (N=1)** | Mean (SD): 70.0 (NA) Median [Min, Max]: 70.0 [70.0, 70.0] Missing: 0 (0%) |
| **Understanding suit limitations (N=1)** | Mean (SD): 95.0 (NA) Median [Min, Max]: 95.0 [95.0, 95.0] Missing: 0 (0%) |
| **Understanding the biomechanics of getting in and out of suit (injury prevention mitigation) (N=1)** | Mean (SD): 15.0 (NA) Median [Min, Max]: 15.0 [15.0, 15.0] Missing: 0 (0%) |
| **Understanding the environment you'll be in, how it impacts your ambulation (N=1)** | Mean (SD): 90.0 (NA) Median [Min, Max]: 90.0 [90.0, 90.0] Missing: 0 (0%) |
| **Understanding where you found the sample and noting that on a map (N=1)** | Mean (SD): 20.0 (NA) Median [Min, Max]: 20.0 [20.0, 20.0] Missing: 0 (0%) |
| **Understanding worksite accessibility—PSRs, slope, terrain features (N=1)** | Mean (SD): 15.0 (NA) Median [Min, Max]: 15.0 [15.0, 15.0] Missing: 0 (0%) |
| **Understanding your personal consumption rate of consumables (N=1)** | Mean (SD): 90.0 (NA) Median [Min, Max]: 90.0 [90.0, 90.0] Missing: 0 (0%) |
| **Understanding your spatial distances (horizon differences from Earth) (N=1)** | Mean (SD): 65.0 (NA) Median [Min, Max]: 65.0 [65.0, 65.0] Missing: 0 (0%) |
| **Where astronaut grew up (N=1)** | Mean (SD): NA (NA) Median [Min, Max]: NA [NA, NA] Missing: 1 (100%) |
